# Implementing a Health and Wellbeing Programme for Children in Early Childhood: A Preliminary Study

**DOI:** 10.3390/nu9091031

**Published:** 2017-09-18

**Authors:** Karen Munday, Megan Wilson

**Affiliations:** School of Nursing, Eastern Institute of Technology, Taradale, Napier 4112, New Zealand

**Keywords:** education programme, fruit and vegetable consumption, ultra-processed foods, kindergarten

## Abstract

In New Zealand, there is a high prevalence of childhood poverty and food insecurity, which can impact a family’s ability to provide high quality, nutrient dense foods for their children. In an attempt to increase the quality of the food consumed by children attending a decile two (low socio-economic) kindergarten and to address food insecurity issues, an educational health and wellness initiative, in conjunction with a free lunch programme, was introduced. The impact of the lunches and the effectiveness of the programme were evaluated. Baseline and end-intervention 24-h modified dietary recall questionnaire data and a vegetable- and fruit-specific food frequency questionnaire (FFQ) were collected. A follow-up FFQ was administered six months after the end of the intervention. The nutrient composition of the foods recorded in the 24-h recall questionnaires were analysed using FoodWorks8™. Whilst no significant differences were observed with the intakes of individual nutrients, there was a significant decrease in the consumption of ultra-processed snack foods (*p* = 0.015). The results of the follow-up FFQ, including the comments collected from the parents, suggested that the intervention had a longer-term positive impact on not only the children involved in the study but also on their *whānau* (wider family members)

## 1. Introduction

The high prevalence of childhood poverty and food insecurity detrimentally impacts food availability in many low socio-economical households in New Zealand [1]. Research suggests that those with food insecurity issues are more likely to select energy-dense diets, high in refined grains, added sugars, and fat, and are less likely to purchase fresh, nutrient dense foods [2]. The New Zealand Ministry of Health (MOH) recommends that infants and children should eat at least two servings of vegetables, increasing to three servings at aged five, and two servings of fruit each day [3]. The 2002 New Zealand National Nutrition Survey (Children) showed that only two out of five children, aged five to fourteen years, ate the recommended daily servings of fruit and only three out of five ate the recommended daily servings of vegetables [4]. 

In New Zealand, the decile rating system was set up by the government to determine how much government funding a school receives. Schools are rated on a scale of one to ten based on the socio-economical status of the families sending their children to the school. A low decile rating suggests that the children come from families with a low socio-economical status. Staff at a decile two kindergarten in New Zealand noticed that, despite the kindergarten’s healthy eating guidelines, the children’s packed lunches typically contained high proportions of highly processed, nutrient-poor snack foods. Additionally, they were aware that parents were unwilling to send their child to the kindergarten if they had insufficient food at home to provide a packed lunch for them, thus food insecurity was impacting on the child’s opportunity to attend kindergarten.

In an attempt to increase the quality of the food consumed by the children and to address the food insecurity issues, staff decided to provide free lunches for the children. In addition to the free lunches, and in collaboration with the local university, the kindergarten also instigated a range of initiatives to encourage healthier eating habits in the children and their families. The drive to improve eating habits was based on the understanding that setting up healthy eating patterns during childhood could have positive health benefits that last throughout life [5]. One of the main aims of the lunches and the educational intervention was to increase the consumption of vegetables, fruit, and dairy and to decrease the consumption of highly processed, shop-bought snack foods. Diets high in vegetables and fruits, in conjunction with lowered saturated fat and those that are low in ‘ultra-processed foods’ (foods with raw or minimally processed foods in their composition [6]) are link to a decreased risk of developing heart disease and other chronic diseases [7,8,9]. The head teacher at the kindergarten wished for the effectiveness of the programmes to be evaluated so they could decide whether to continue the lunches on a permanent basis. Additionally, they wanted to trial the educational package to be able to provide feedback to other early childhood centres about how the different aspects of it were received by the children and how the staff managed to incorporate different activities into the kindergarten routine. Following the ten week intervention, the teaching plans and information about useful resources were made freely available to other pre-schools.

There have been numerous interventions trialed in pre-school environments that have been successful in positively influencing food intake [10,11,12]. As the kindergarten had the resources available to try a number of different methods to improve the dietary habits of the children, a multi-faceted approach was trialed. The intervention described in this paper was based on the findings of a number of successful interventions run in similar environments. 

A major component of the intervention involved teaching the children about the benefits of eating fruits and vegetables. This has been shown to have a positive impact on consumption. A comprehensive review by Rasmusses et al. [13] investigated potential determinants of fruit and vegetable intake in children and adolescents aged 6 to 18 years. Whilst the greatest influences on fruit and vegetable consumption were non-modifiable factors such as gender (girls tended to eat more fruits and vegetables than boys) and age (with younger children eating more than older children), knowledge was identified as a modifiable factor that increased consumption. Education has also been shown to have a longer-term impact on dietary habits. Céspedes et al. [14] discussed the impact a preschool-based educational intervention aimed at promoting cardiovascular health had on knowledge, attitudes, and habits relating to healthy eating and activity levels 36 months after its completion. The follow-up study showed that the positive dietary changes and increased knowledge about the cardiovascular system were still apparent. 

Additionally, involving children in food preparation can encourage them to eat vegetables. The kindergarten already had a vegetable plot, but, prior to the intervention, it was not being fully utilized. A study by van der Horst [15], involving 47 primary-school aged children from Lausanne, Switzerland, concluded that when children were involved in preparing their own food, they increased their consumption of vegetables. A Canadian study, again looking at primary school-aged children, showed that those children who had a greater involvement in food preparation made healthier food choices than children who were involved less in meal preparation activities [16]. The kindergarten also tried to involve the parents and the children’s wider families (*whānau*) in looking after the vegetable plot. A review by Hingle et al. [17] concluded that, whilst there were limited data to show the benefits of parental involvement in changing the eating habits of younger children, studies that directly involved parents were more likely to show positive or mixed results when compared to those that did not. Better nutrition education for parents and members of the wider family has been shown to be important in increasing the level of nutritional intake in children [18,19]. The kindergarten offered *whānau* cooking classes to show parents how to cook healthy meals on a budget.

Another strategy that has been shown to increase fruit and vegetable consumption is to offer children a variety of fruits and vegetables [20]. Whilst forcing the consumption of foods during childhood can have a negative impact on an adult’s eating habits [21], short-term repeated exposure of a given food can increase food preferences [22] and influence adult dietary patterns. Providing repeat exposure to new fruits and vegetables has also been shown to increase the likelihood that children will try and then accept new foods [23]. For this reason, another component of the intervention involved the frequent offering of numerous different fruits and vegetables to the children. These tasting sessions were designed to allow the children to try a variety of different fruits and vegetables in a non-threatening environment. 

The above strategies, including a formal education programme plus fruit and vegetable tasting sessions, the encouragement of the children to grow and cook with vegetables, and healthy eating cooking classes for parents and *whānau* were the key components of the intervention. The aim of the research was to assess the impact of the free lunches, in conjunction with the other initiatives, on the short-term dietary intakes and longer-term dietary habits of the children enrolled in the kindergarten.

## 2. Materials and Methods

The intervention was undertaken in a low decile kindergarten situated in Palmerston North, New Zealand. The children attending the kindergarten attended up to five days per week for between four and five and a half hours per session. They were aged between three years eight months and five years one month, with an average age of four years two months at the start of the study.

Ethics permission was obtained from the Research Ethics Approval Committee at the Eastern Institute of Technology (reference number 18/14, dated 30 June 2014). The parents/*whānau* of the 32 children enrolled at the kindergarten were contacted and asked if they wished to provide consent for their child or children to be part of the study. The requirements for participation were explained, and consent was obtained.

Convenience sampling was undertaken to recruit the study participants. The parents/*whānau* of all the children in the kindergarten were invited to enroll their children in the study, but only seventeen of the thirty-two children enrolled at the time received consent to participate in the study. Seven of these children were lost following the completion of the study so no follow-up data were collected from them.

Demographic and dietary data were collected primarily through interviewing the parents, with some dietary data collected by the kindergarten staff. Information relating to the tasting sessions was collected by the students running the sessions. Demographic data were collected at baseline, including age, gender, height, weight, the country of birth of the child and the parents, the educational status of parents, the first language of the parents, the number of household members, and household income. 

Whilst all the children in the kindergarten were exposed to all aspects of the intervention, including the free lunches, data were only collected from those whose parents had consented to it. The intervention was run during term three (mid-July to the end of September). The various components of the intervention were bi-weekly education sessions, bi-weekly ‘tasting’ sessions, sticker reward charts, increased utilisation of the kindergarten vegetable plot, and *whānau* cooking classes. The tasting and education sessions were run by second and third year Massey University students studying Human Nutrition, under the guidance of a New Zealand Registered Nutritionist.

Tasting sessions were run twice a week. During these sessions, the children gathered together whilst the students talked briefly about the fruits and vegetables that were available for tasting. Four to seven different fruits and vegetables were presented to the children at each session and the children were offered small pieces of each to try. Each time a child tasted (licked, put in their mouth, or ate) a piece of fruit or vegetable, they were rewarded with a colour-coded sticker that they were able to stick on a sticker chart, indicating which fruit or vegetable they had tried. This enabled the results of the tasting sessions to be collated. Initially, the foods were presented on one large plate, which was offered to the children in turn. It was noted that there was a gradual decline over the weeks in the number of fruits and vegetables being tried so a new strategy was introduced whereby children were given individual plates with the chopped up pieces of fruit and vegetables on them. The results of the tasting sessions were reviewed to look at the habits of the individual children and to determine if any discernible patterns could be seen.

The formal education sessions were approximately half an hour long and covered subjects ranging from the introduction of the concept of ‘healthy’ food to discussions regarding where fruits and vegetables come from and their health benefits. A variety of strategies, including stories, songs, DVDs, colouring-in, and growing their own plants from seeds, were adopted. In addition to the formal teaching sessions, a ‘healthy eating environment’ was set up at the kindergarten with books and wall displays promoting the healthy eating message, as well as encouragement for the children to be involved in tending the kindergarten’s vegetable plot.

Baseline modified 24-h recall questionnaires were completed for each of the subjects on three separate occasions during the last week of term two. The modification involved the dietary questionnaires being completed by the kindergarten teachers during the time when the children were at the kindergarten, directly following each meal occasion. The researcher drew up a roster so the teachers were aware of which children required a dietary questionnaire on which day. The researcher contacted the parents the following day to complete the remainder of the questionnaire. It was decided to only collect data on weekdays rather than weekends as part of the objectives of the study was to assess the impact of the supplied lunches on eating habits. Three further 24-h recall questionnaires were administered during the last two weeks of the study, weeks nine and ten of term three. The results of the modified 24-h recall questionnaires were analysed using FoodWorks8™ (Xyris Software Pty Ltd, Kenmore Hills, Australia). The distribution of the data was checked, and two-tailed *t*-tests were performed using Microsoft Excel to determine if there were any significant differences in nutrient intake between the baseline and end-intervention time-points. The consumption of fruits and vegetables by meal occasion was also investigated, as was the number of ultra-processed foods consumed prior to the intervention and during the last two weeks of it. The definition of ultra-processed foods was based on the definition provided by Monteiro et al. [24] in which the characteristics of ultra-processed foods were defined as being ‘typically energy dense; have a high glycaemic load: are low in dietary fibre, micronutrients, and phytochemicals: and are high in unhealthy types of dietary fat, free sugars, and sodium’ [24] (p. 14). For the purpose of this study, the foods considered ‘ultra-processed’ included shop-bought packaged snack foods such as ‘chippies’ (potato crisps), cookies and biscuits, chocolate bars, sweet bakery products (such as muffins and donuts), fruit rolls or roll-ups, ice-creams, and sugar-sweetened beverages (‘fizzy’). However, the classification of ‘ultra-processed foods’ by Monteiro et al. [24], taken from the Euromonitor Passport Global Market Information Database, also included all forms of processed meats and meat products, including sausages, sausage rolls and pies; pizza; breakfast cereals; bread; and flavoured yoghurts. These were not counted as ‘ultra-processed’ foods in this study as a differentiation was made between processed foods that were deemed ‘staple’ items (such as bread, cereals, meat-based products, and dairy) versus foods that children were provided as a ‘treat’ (such as chippies, cookies, and fizzy drinks).

A food frequency questionnaire, which focused on intakes of fruits and vegetables and dairy products, was administered during the first week of the study. The same food frequency questionnaire was administered again six months following the end of the intervention. At this time-point, the researcher asked an open-ended question about whether the parent has noticed any difference with their child’s eating habits since taking part in the dietary intervention. Some of the parents made comments without being questioned. The comments were documented. The food frequency questionnaires were analysed to determine if there were any changes in the variety or frequency of fruit and vegetables consumed at the beginning of the intervention and six months following the completion of it.

Attendance data were collated from the attendance records, and a comparison was made between the attendances in term two and term three.

## 3. Results

### 3.1. Demographic Data 

The percentage of boys and girls enrolled in the study did not reflect the percentage that were enrolled in the kindergarten. At the start of the intervention, the kindergarten had 56% males (*n* = 18) and 44% females (*n* = 14). Only four females were enrolled in the study (24%) as opposed to thirteen males (76%). 

The average age of the children was four years and two months. According to the New Zealand Ministry of Health growth charts (2010) and the weight-to-height body mass index (BMI) conversion chart, none of the children were obese, but four of the boys and one of the girls were overweight. Sixty nine percent of the boys were below average height for their age, with seven of them (54%) were below the 25th height for age centile (Table 1). Only one of the four girls was below average height for her age.

Sixteen families were involved in the study, with one family having twins enrolled at the kindergarten. Two of the seventeen children were born outside of New Zealand, and eight of their parents were born overseas. The educational status of the parents ranged from high school qualified (*n* = 11) to post-graduate level (*n* = 3), with a further 15 having undertaken tertiary education, including three with post-graduate qualifications. For the 14 families who disclosed their income, the annual income ranged from less than $20,000 per annum (*n* = 1) to $70,000 to $100,000 per annum (*n* = 3). Two families report an income of $20,000 to $30,000 per annum, and four families fell within the $30,000 to $50,000 income range. The number of people per household ranged from two to eight, with an average of five.

### 3.2. Modified 24-h Dietary Recall Questionnaire

Following the Foodworks8™ analysis, two-tailed paired *t*-tests were performed on a selection of the nutrients to determine if there were any significant differences between the two time points (Table 2). No significant differences were observed between the two time-points for any of the nutrients analysed. 

### 3.3. Consumption of Snack Foods

The consumption of ultra-processed snack foods was determined from the modified 24-h recall questionnaires. The number of ultra-processed foods documented as being consumed at baseline by the 17 study participants was documented as being 101. This fell to 63 items during the end-intervention data collection period (*p* = 0.015). The majority of the decrease came from snack foods consumed whilst the children were at the kindergarten, which decreased from 48 at baseline to 22 during the data collection period at the end of the intervention.

### 3.4. Attendance

In order to determine whether the free lunches impacted on the level of attendance at the kindergarten, weekly attendance data from term two and term three were compared using a two-tailed paired *t*-test. There was no significant difference detected between the level of attendance over the two terms (*p* = 0.983).

### 3.5. Food Frequency Questionnaire

Food frequency questionnaire data were collected for all seventeen children during the first week of the intervention. The parents of ten of the children were contactable six months after the completion of the intervention. All the children ate fruit at the beginning of the intervention, but one child did not eat vegetables. At the six month follow-up period, this child was regularly eating four different vegetables. At the follow-up time-point, eight of the ten children had increased the variety of fruits that they ate, and eight had increased the variety of vegetables. One child almost doubled the variety of vegetables that they consumed, increasing from 17 at baseline to 30 at follow-up. 

## 4. Discussion

The kindergarten intervention was multi-faceted and included formal education sessions, tasting sessions, and *whānau* cooking classes run in conjunction with the introduction of a free lunch programme. The children were also encouraged to cook with and consume vegetables grown in the kindergarten vegetable plot. The results of the food diary analyses showed that the children consumed significantly fewer ultra-processed foods at the end-intervention time point compared to at base-line.

The decile rating of the kindergarten indicated a high level of economic deprivation among the families of the children attending it. According to the New Zealand Income Survey for the June 2014 quarter [25], the median weekly income for income from all sources was $600. This equated to an annual income of $31,200. The income data showed that ten of the families had annual incomes of more than $50,000, which was considerably higher than the New Zealand median. The self-selection process may not have been so successful at capturing the children from the lower-income families. There was no pattern observed between income status and number of household members. 

It was unclear why there was a bias towards more boys being enrolled in the study than girls. There is no suggestion in the literature that one gender has a higher prevalence of ‘picky eaters’ than the other [26], thus it is unlikely that the parents would chose to enroll their children based on how they perceive how they would respond to the introduction of new foods.

The demographic data showed that more than half of the boys (54%) fell below the 25% height for age centile, with one of the boys falling on the 2nd height for age centile, indicating a moderate level of stunting. Whilst some ethnicities are typically of shorter in stature, only one of the boys was of non-New Zealand descent, hence this was unlikely to account for the disproportionate number of the boys who had low height for their age. Impaired growth during early childhood can be the result of poor dietary intakes, linked to food insecurity. An analysis of the Mexican National Health and Nutrition Survey results (2012) showed that stunting was more prevalent in children that came from homes with moderate or severe food insecurity than in those that lived in homes with only mild or no food insecurity issues [27]. 

The level of attendance at the kindergarten did not differ between term two and term three. Attendance during term two ranged from between ninety three percent in week three, dropping to sixty eight percent in week seven. In term three, the highest attendance occurred in week five (eighty eight percent), falling to sixty nine percent in week six. July is typically the coldest month of the year, with both June and July having the highest amount of rain. Asthma and respiratory tract infections are associated with cold, damp housing and disproportionately affect those living in the most socio-economically deprived areas [28]. Typically, absences from the kindergarten are highest during the winter months.

A significant difference in eating patterns was observed between the pre-intervention and the end-intervention time points with a reduction in the consumption of ultra-processed snack foods. With the introduction of the free lunches, children were no longer required to bring food from home. Prior to the introduction of the free lunches, parents were informed of the kindergarten’s dietary guidelines, but staff observed that these were seldom followed. When confronted by the kindergarten staff, parents often justified the inclusion of highly processed ‘treat’ foods in their children’s lunch boxes through saying that they did not want their children to be singled out or feel that they were missing out for not having these foods. The introduction of lunches meant that all the children were offered the same food at the kindergarten, thus removing the pressure on the parents to provide their children with packaged ‘treat’ foods. 

The analysis of the dietary data did not show any significant changes in nutrient intakes. There was a high degree of variance in the daily intakes of the different nutrients. The nature of the recruitment of the study participants (resulting in only 17 participants from a total pool of 32 children) meant that the study possibly lacked the power to be able to detect any significant differences in nutrient intakes. According to the Ministry of Health’s (MOH) Nutrient Reference Values (NRV) for Australia and New Zealand [29], the average energy intakes fall within the range that would be deemed acceptable for children within the age-group of the cohort being studied. The Estimated Average Requirement (EAR) for calcium is 360 mg per day for children between one and three years old and 520 mg for those between four and eight years old [29]. Whilst nine of the children consumed, on average, more than 520 mg of calcium a day at baseline, three individuals had average intakes of less than 300 mg per day. The lowest individual average intakes of calcium were 233 mg, 278 mg, and 294 mg, which increased to 332 mg, 500 mg, and 484 mg, respectively, at the end-intervention time-point. The increase in calcium could be attributed to the increase in yoghurt consumption by these children.

The adequate intake level of fibre for children aged four and eight years is 18 g per day [29]. Intakes of fibre were, in general, very low across this cohort of children. The child with the lowest average intake at baseline (8.5 g) still had the lowest average intake (5.9 g) at the end-intervention time-point. Only three children consumed, on average, more than 18 g of fibre at both of the time-points (and two others consumed over 18 g at one of the time-points). Fruits and vegetables are good sources of fibre. The fact that an individual’s fibre intakes were relatively constant between the two time-points reflected a lack of an increase in the quantity of fruits and vegetables consumed during the intervention period. There are potentially two reasons as to why the food diary data did not show an increase in fruit and vegetable consumption, nor any changes in the nutrient intakes of the children. Firstly, this could have been a result of the selection process missing the children who could have potentially benefited the most from the lunch intervention. The high intake of snack food and the low intake of fibre, one of the components of the recently developed Health Dietary Habits Index [30], suggested that the children included in the study did eat relatively poor quality diets at baseline. It is therefore unlikely that the lack of a change in nutrient intakes was a result of the self-selected cohort of children not requiring any improvements in their diets. Secondly, there was a big discrepancy between the lunches that the children were recorded as having eaten during the intervention and the lunch menu that was set up at the beginning of the study. The lunch menu included a wide variety of sandwiches and toasties made with ham, eggs, cheese, beans, sardines, and tuna, and all contained some vegetables. The food diaries showed that on the days during the intervention when the food recall questionnaires were completed, the children only ate jam or marmite sandwiches, made with white bread and with no vegetables or fruit. Had the children eaten the more nutritious lunches, there is a higher probability that a difference in fruit and vegetable consumption, and possibly in dietary nutrients, would have been observed. There was some resistance from the parents to the introduction of the lunches, with parents worrying that their children would refuse to eat the sandwiches/toasties on offer and would go hungry. For this reason, it was agreed that an option of jam or marmite sandwiches would be offered each day. Whether the lack of any other type of sandwich being eaten was a result of this becoming the only option on offer or whether this was a reflection of the children’s choice is not clear. 

The results of the tasting sessions were collected on a sticker chart, which was also used as an incentive to encourage the children to try the novel fruits and vegetables. The literature suggests that children develop their food habits during the first six years of life [31], and repeated exposure to new foods increased the likelihood that children will try them. It has been suggested that younger children may require up to fifteen exposures to a new food before they are willing to accept it [32]. The data collected from this study showed that the willingness to taste the fruits and vegetables increased after the way they were presented was changed from offering them on a platter to giving each child their own plate of fruit and vegetables. Other than this, there was no apparent trend in their willingness to try new foods as the intervention progressed. All the children in the study tried at least one piece of fruit or vegetable. The tasting sessions involved the introduction of a wide variety of fruits and vegetables; some of them were only offered on a single occasion. It may have been more beneficial to offer a smaller selection of fruits and vegetables multiple times over the ten week intervention period.

The uptake of the *whānau* cooking class was disappointingly low. A large amount of thought and effect was put into these classes, including the recruitment of a local chef to run them and the provision of childcare during the sessions. Whilst the time of the classes was cited by some of the parents as a reason why they did not attend (they were held straight after the kindergarten session had finished), generally there was a lack of interest from the parents.

The food frequency questionnaires were completed during the first week of the study and again six months after its completion. The purpose of the food frequency questionnaire was to document the variety of fruits and vegetables eaten by children and the frequency at which they ate them. The researcher only managed to collect follow-up food frequency questionnaire data on ten of the seventeen subjects. Whilst all the children ate fruit at the beginning of the intervention, one child did not eat any vegetables. At the six month follow-up period, this child was regularly eating four different vegetables. At the follow-up time-point, eight of the ten children had increased the variety of fruits that they ate, and eight had increased the variety of vegetables. One child almost doubled the variety of vegetables that they consumed, increasing from 17 at baseline to 30 at follow-up.

When collecting the follow-up food frequency questionnaire data, the researcher asked the parents a very general question about whether they had noticed any other differences in their child’s eating habits since they had taken part on the intervention. All bar one of the parents had a positive response to this question or offered a comment without being prompted. There was not sufficient data to perform a thematic analysis on the comments, but there were some common themes that emerged. These themes included that the children had an increase willingness to try new foods (not just fruit and vegetables), that there was a decrease in food waste so parents were more willing to buy fruits and vegetables, and that others in the family were positively influenced by the child’s improved eating habits. 

As there was no control group, it was not possible to determine if specific aspects of the programme impacted on the children’s dietary habits. It was deemed unethical to exclude a group of kindergarten children from the free lunch programme, and the practicalities of the way that the kindergarten ran meant it was not possible for one group to be excluded from the education programme. Running the lunch programme at a different low decile kindergarten but without the educational component was considered as an alternative approach. However, due to lack of funding and resources, it was decided to initially run the intervention at a single kindergarten with the view of transferring the programme to other kindergartens should improvements in eating habits be observed. This would allow for the study to be repeated with a control arm. A recently published comprehensive review [33] concluded that the involvement of children in school gardening programmes appeared to be more beneficial in changing eating habits than nutrition education; there is some evidence that taste lessons decrease neophobia (the fear of trying new foods), at least in the short-term; repeat exposure to a variety of fruits and vegetables is effective at increasing children’s intake; and making fruit and vegetables available for children appears to be an effective strategy to increase intake. 

The small number of children who received consented to take part in this study and the number of participants who were lost during the follow-up phase made it difficult to draw robust conclusions about the impact that the intervention had on the children’s eating habits. 

Another major limitation of the study was the self-selection of the participants. The demographic data, in particular the income brackets of the parents, suggest that the parents who volunteered for their children to be part of the study were not necessarily representative of all the families with children at the kindergarten.

## 5. Conclusions

The results of this preliminary study indicate that there is the potential, using a multifaceted health and wellbeing programme, to positively impact on the dietary habits of young children. In order to build on the findings of this study, the suggestion would be to repeat the study using a greater number of participants and with the inclusion of a control group. 

The introduction of the free lunches at the kindergarten had a significant impact on the amount of highly processed packaged foods consumed by the children. The nutrient intake of the children did not change between the pre-intervention and end-intervention time-points, indicating that the programme did not succeed in encouraging a significant increase in fruit and vegetable consumption in the short-term. However, the follow-up data, collected six months following the end of the intervention, suggested that the intervention did have a positive impact on the dietary intakes of fruit and vegetables or the children’s general eating habits. One theme that emerged from the comments was that the children were more willing to try new foods following the intervention; plus parents were more willing to buy fruits and vegetables for their children if they were confident that they would be eaten rather than wasted. Another common comment was that the changes in the child’s dietary habits positively influenced other family members and *whānau*.

It became apparent during the intervention period that the amount of time required to prepare nutritious lunches on a daily based placed too big a burden on the kindergarten staff, impacting on their ability to carry out their regular duties. In order for a similar lunch programme to be successful in the future, it would be necessary to have the finances to provide both the food and personnel to prepare and serve the food. There would also need to be acceptance from the parents as one of the barriers faced by this kindergarten was the lack of support and interest from the children’s families.

Whilst the formal educational component was labour intensive, the staff found it easy to promote the ‘healthy eating’ message in the kindergarten. They did so through a variety of mediums, including the provision of books, wall displays, and activities, and through songs and stories. This is something that could be easily incorporated into other early childhood centres.

## Figures and Tables

**Table 1:** Baseline demographics of the study participants.

Gender	BMI-For-Age Classification (*n* (%))		Height Centiles				Age at Baseline (years and months)
	Normal Weight *	Over-Weight *	>25th	25th to 49th	50th to 75th	<75th	
Male (*n* = 13)	9 (70%)	4 (30%)	7 (54%)	2 (15%)	3 (23%)	1 (8%)	Mean = 4.2
							Range = 3.9 to 5.1
Female (*n* = 4)	3 (75%)	1 (25%)	0	1 (25%)	1 (25%)	2 (50%)	Mean = 4.2
							Range = 3.8 to 4.7

* Based on the New Zealand Ministry of Health weight-to-height body mass index (BMI) conversion chart.

**Table 2:** Nutrient composition of baseline and end-intervention diets (*n* = 17) and Estimated Average Requirements (EAR).

Nutrient	EAR (per day) (1–3 years/4–8 years)	Baseline Mean (SD)	End-Intervention Mean (SD)	*p*-value
Total energy (kJ)		5492 (1309)	5281 (1224.7)	0.460
Protein (g)	12 g/16 g	47 (13.0)	49 (16.2)	0.417
Carbohydrate (g)		177 (46.1)	164 (37.3)	0.176
Fat (g)		45 (13.1)	43 (12.1)	0.574
Saturated fat (g)		20 (6.8)	19 (7.0)	0.774
Fibre (g)	14 g/18 g *	15 (5.4)	15 (5.0)	0.636
Vitamin C (mg)	25 mg	67 (33.4)	50 (28.7)	0.144
Folate (μg)	120 μg/160 μg	399 (299.7)	382 (197.8)	0.779
Vitamin A (retinol equivalent) (μg)	210 μg/275 μg	291 (194.9)	354 (204.1)	0.226
Sodium (mmol)	9–17 mmol/13–26 mmol *	80 (20.5)	81 (20.1)	0.782
Calcium (mg)	360 mg/520 mg	526 (198.4)	608 (196.2)	0.053

* Adequate Intake (Nutrient Reference Values for Australia and New Zealand, 2006).
